# Supplementary material for: ARRDC5 expression is conserved in mammalian testes and required for normal sperm morphogenesis
Source: Nat Commun. 2023 Apr 17;14:2111. doi: 10.1038/s41467-023-37735-y (PMC10110545; doi:10.1038/s41467-023-37735-y)
Supplement: Supplementary file 1 — Supplementary Information [file 41467_2023_37735_MOESM1_ESM.pdf]

ARRDC5 expression is conserved in mammalian testes and required for normal sperm morphogenesis

Mariana I. Giassetti[1,2], Deqiang Miao[1,2], Nathan C. Law[1,3], Melissa J. Oatley[1,2], Julie Park[1,2], LeeLa D. Robinson[1,2], Lisette A. Maddison[1], Miranda L. Bernhardt[1], and Jon M. Oatley[1,2]*

[1]Center for Reproductive Biology, Washington State University, Pullman, WA, USA; [2]School of Molecular Biosciences, College of Veterinary Medicine, Washington State University, Pullman, WA, USA; [3]Department of Animal Sciences, Washington State University, Pullman, WA, USA

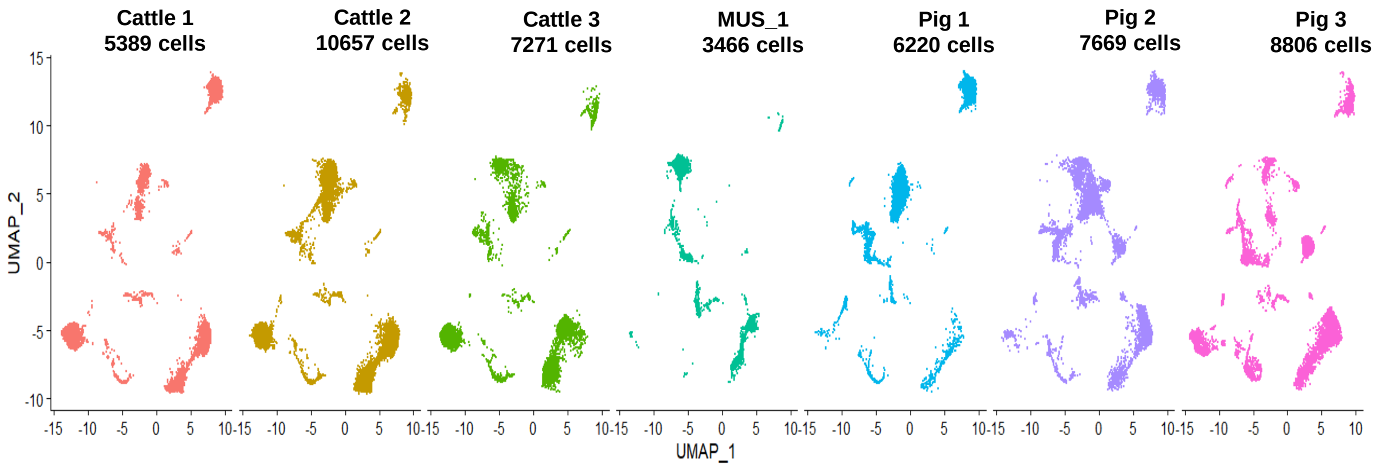

Supplemental Fig. S1: Integrated Uniform Manifold Approximation and Projection (UMAP) plots for single cell RNA-seq analysis of testicular tissue from pre-pubertal cattle, pigs, and mice (MUS).

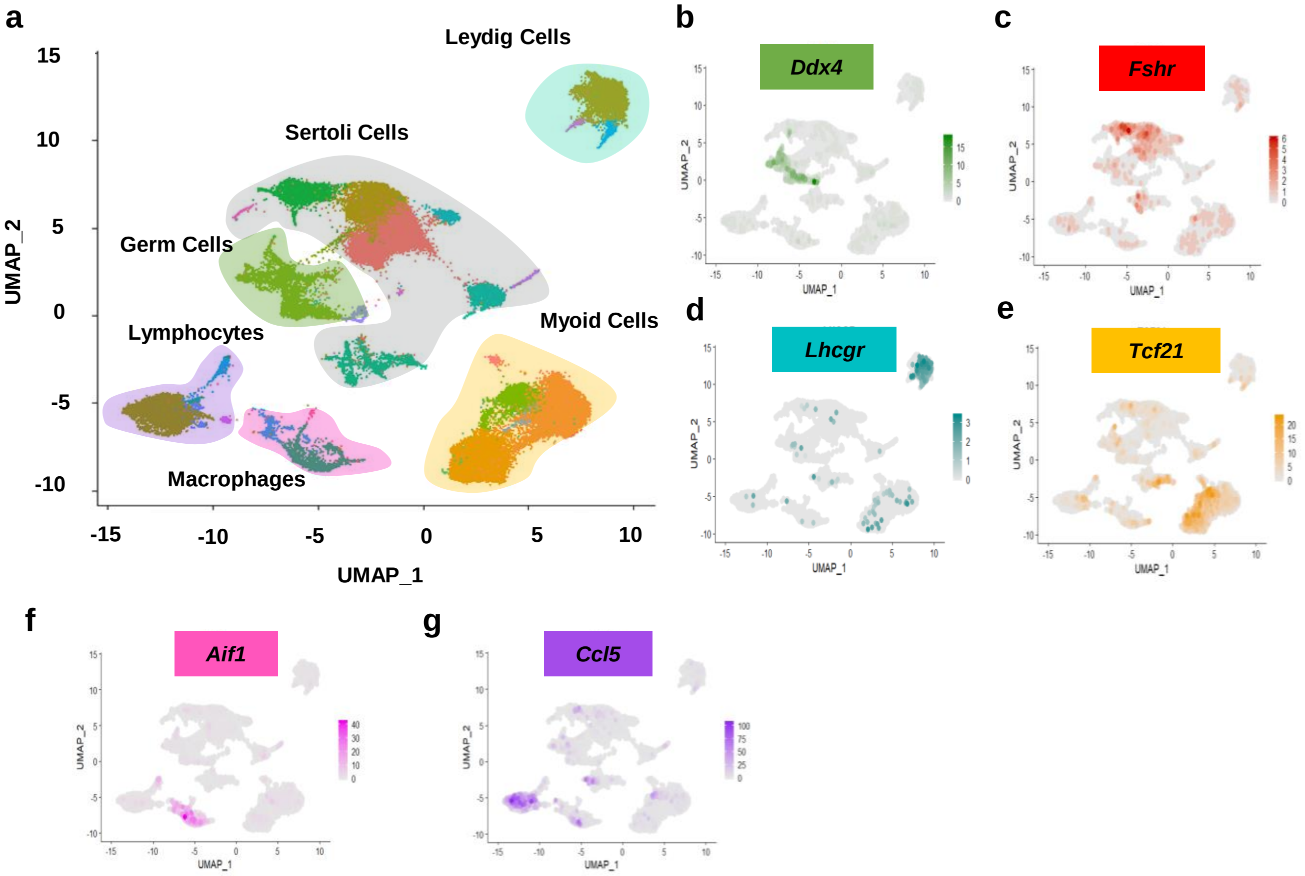

Supplemental Fig. S2: Biomarker analysis to assign cell-type identities to Integrated Uniform Manifold Approximation and Projection (UMAP) clusters. a Full UMAP plot of multispecies integrated single cell RNA-seq analysis with cell cluster identities. **b** Biomarker assessment for germ cell identify genes. **c** Biomarker assessment for Sertoli cell identity genes. **d** Biomarker assessment for Leydig cell identity genes. **e** Biomarker assessment for peritubular myoid identity genes. **f** Biomarker assessment for macrophage identity genes. **g** Biomarker assessment for lymphocyte identity genes.

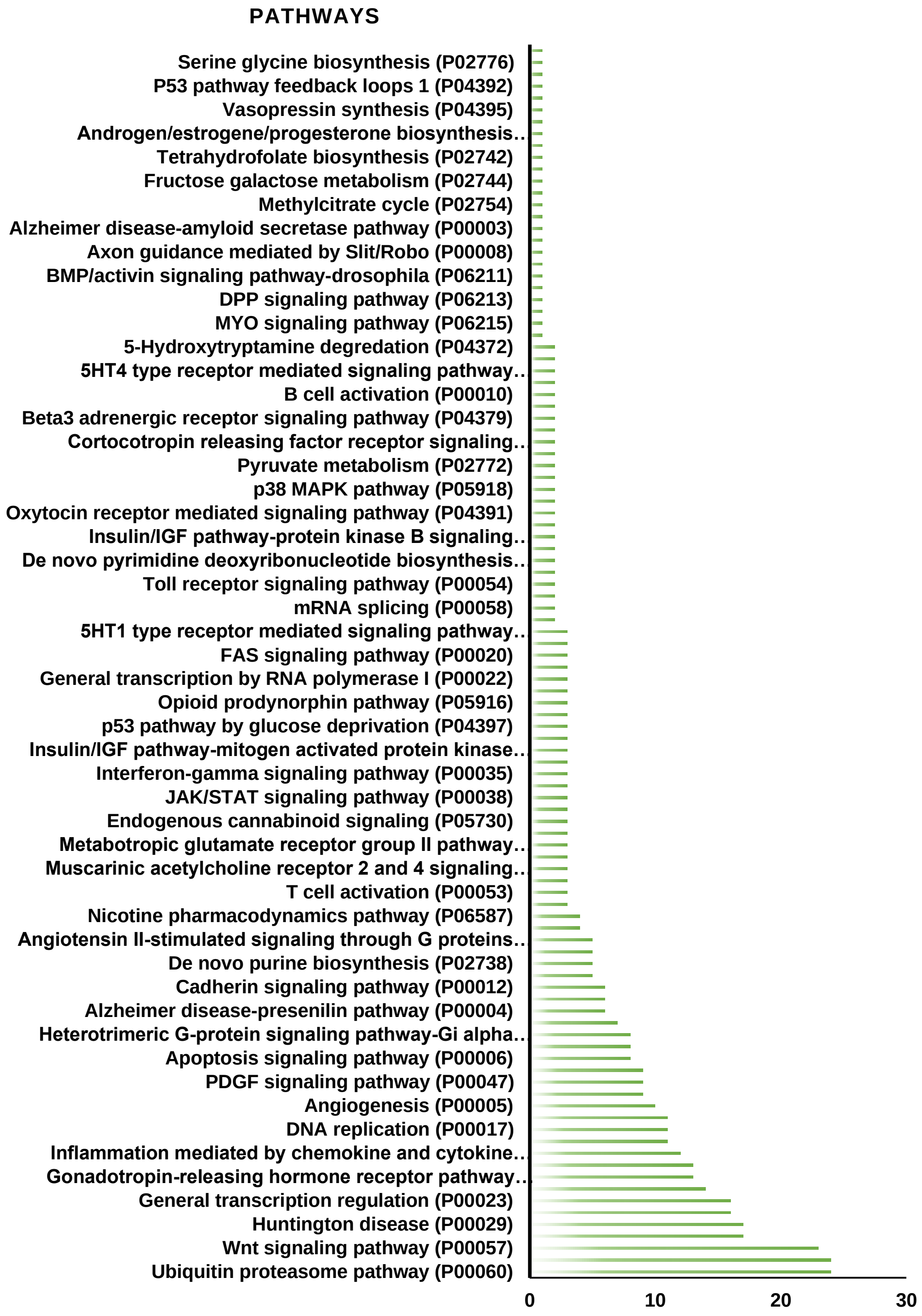

Supplemental Fig. S3: Gene ontology analysis for 10,183 genes commonly expressed in germ cells of cattle, pigs, and mice.

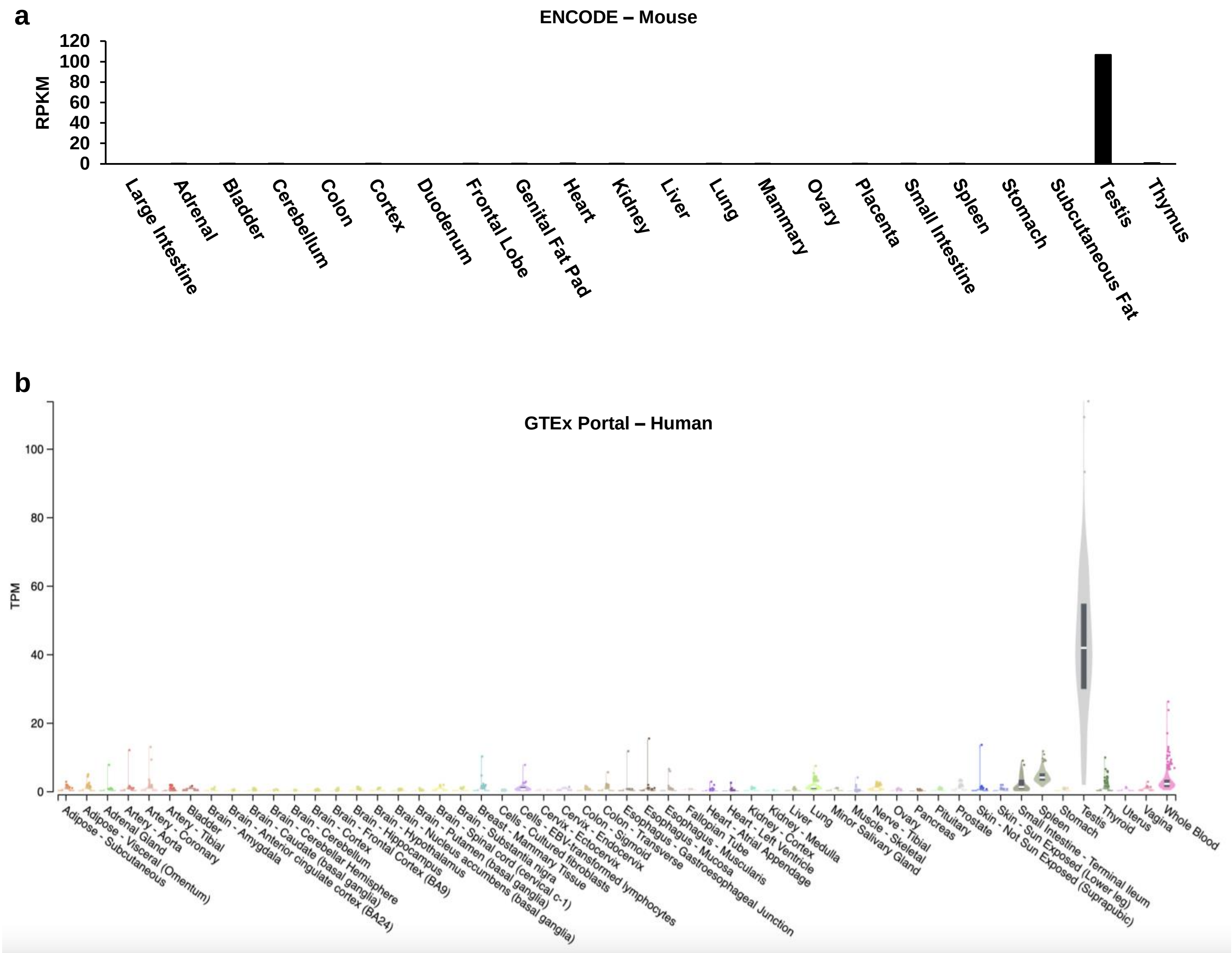

Supplemental Fig. S4: *Arrdc5* gene expression profile in mice and humans. a Graphical representation of data for various mouse tissues downloaded from the ENCODE consortium database. **b** Graphical representation of data for various human tissues downloaded from the GTEx portal.

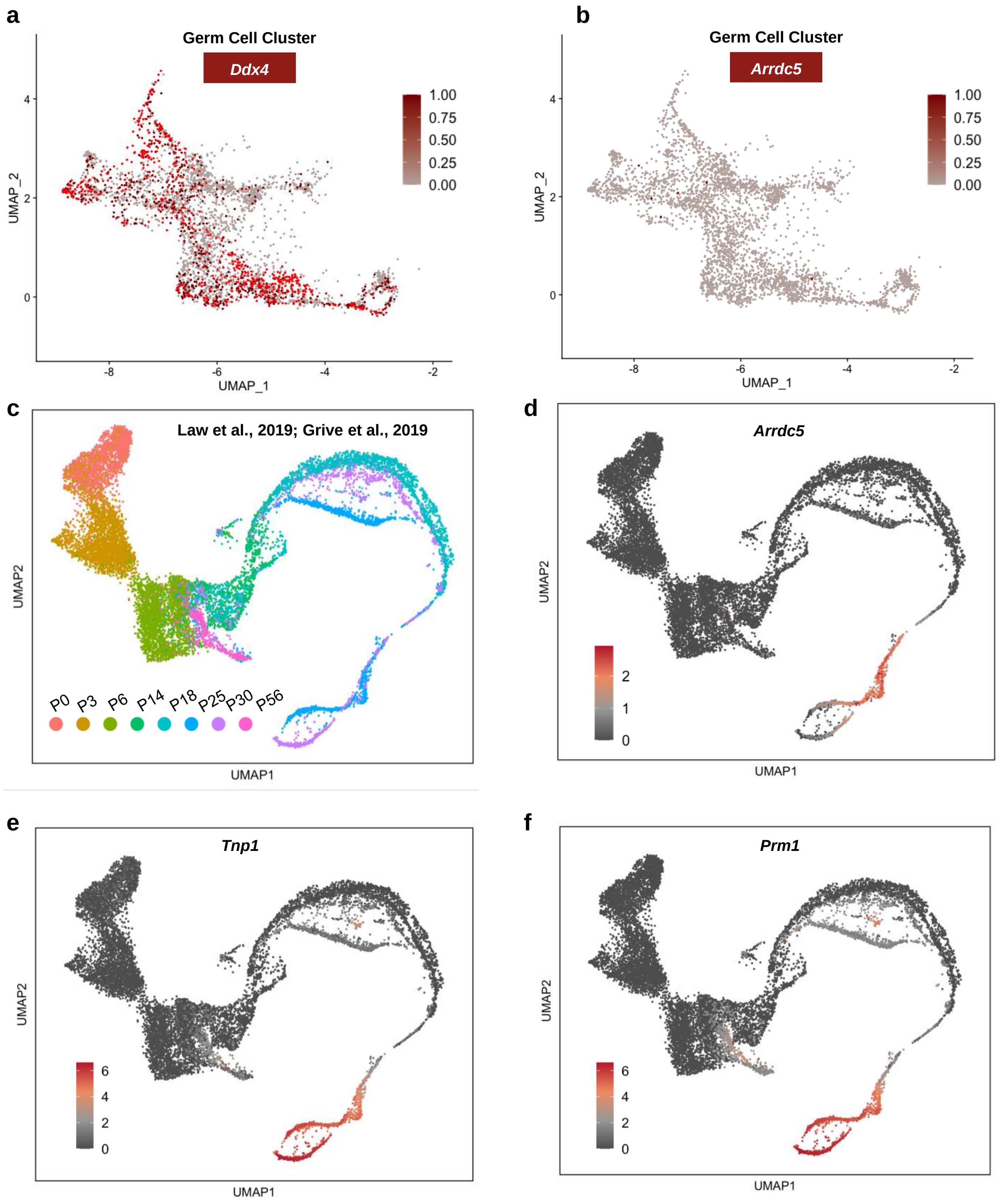

Supplemental Fig. S5: *ARRDC5* gene expression at the single cell level in testes of mice from late prepubertal development through adulthood. a and **b** Uniform Manifold Approximation and Projection (UMAP) plots of the germ cell cluster from the multispecies integrated single cell RNA-sequencing database produced in this study. **c-f** UMAP analysis of mouse testicular germ cells from late prepubertal development (P14) to adulthood (P56) showing total gene expression **c**, *Arrdc5* expression **d**, and expression of the late spermatocyte/spermatid biomarkers *Tnp1* **e** and *Prm1* **f**. Plots were created from data published by Law et al., 2019 and Grive et al., 2019.

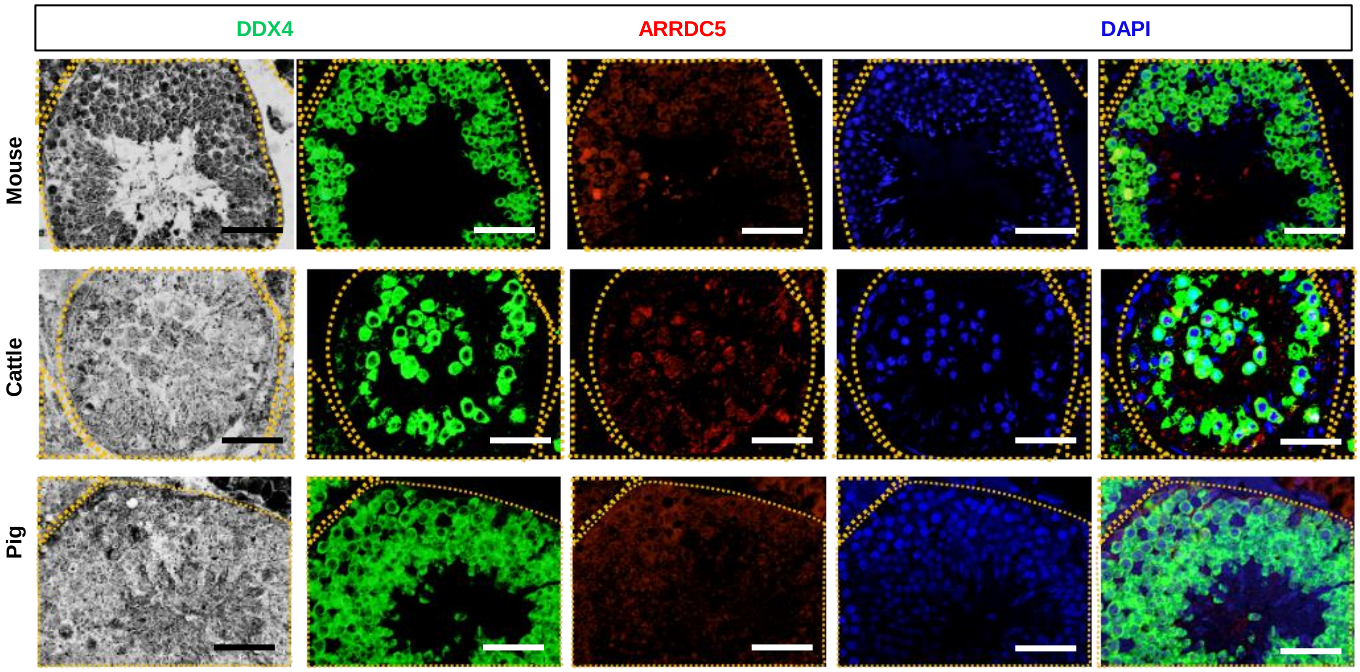

Supplemental Fig. S6: Fluorescent immunostaining for ARRDC5 protein expression in cross-sections of testes from adult mice, cattle, and pigs. Germ cells were labeled with an antibody to the canonical biomarker DDX4 and DNA was stained with DAPI. A commercially sourced antibody for ARRDC5 was used but did not yield specific staining. Bars are 50 μm. Images are representative of 3 independently repeated experiments.

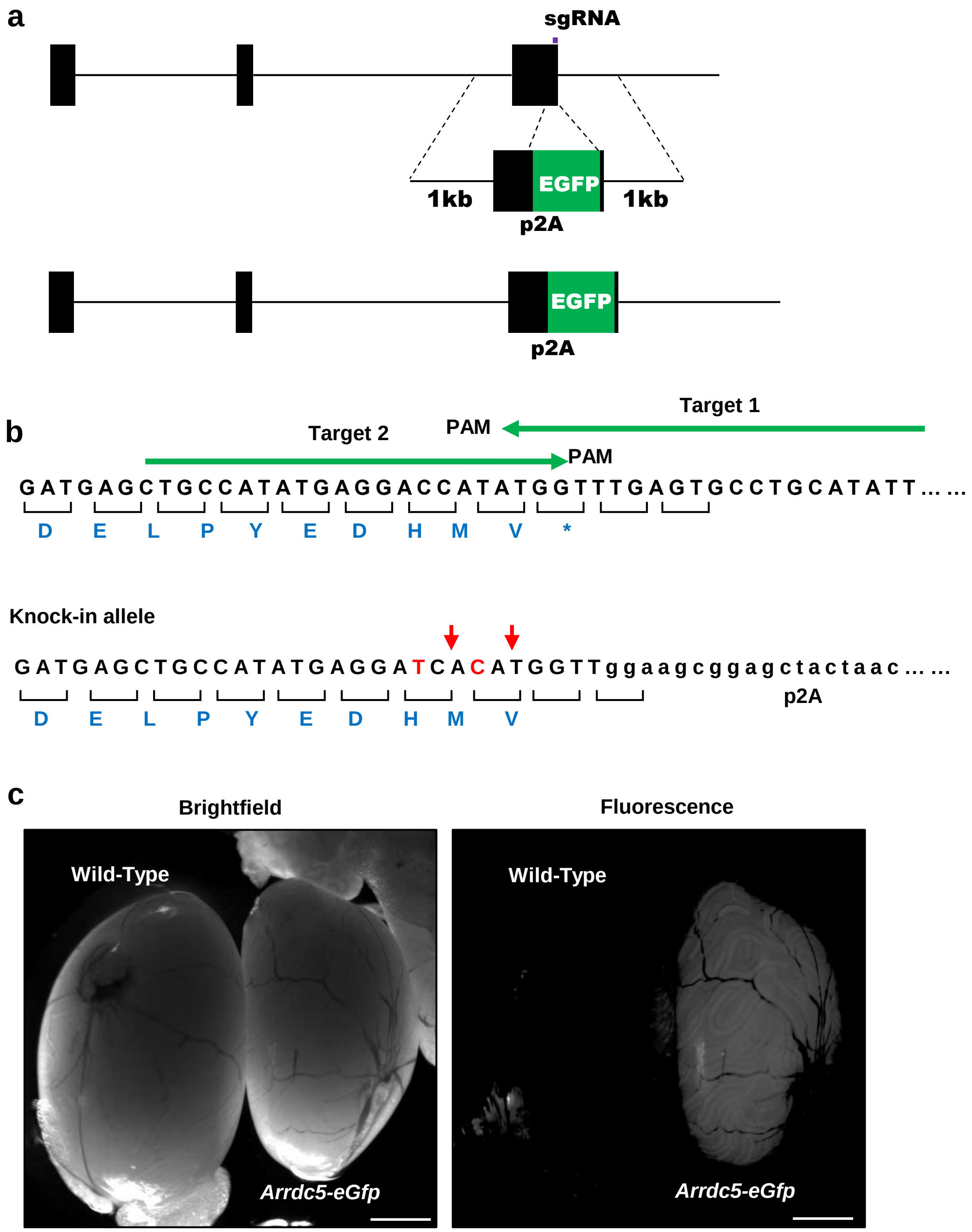

Supplemental Fig. S7: Generation of an *Arrdc5-eGfp* knock-in mouse model. a Schematic of *Arrdc5* gene structure with CRISPR-Cas9 targeting strategy for exon 3. Homology arms of 1kb were used in the repair template. **b** Schematic of positions of Cas9 target sites with target 1 overlapping the stop codon. Two silent mutations were made in the knock-in allele to eliminate Cas9 attack of the donor or modified allele. These mutations in the seed sequence of target 2 were sufficient to avoid Cas9 cleavage. **c** Wholemount testes from adult wild-type and *Arrdc5-eGfp* knock-in mice. Images are representative of 5 independently repeated experiments. Bars are 500 μm.

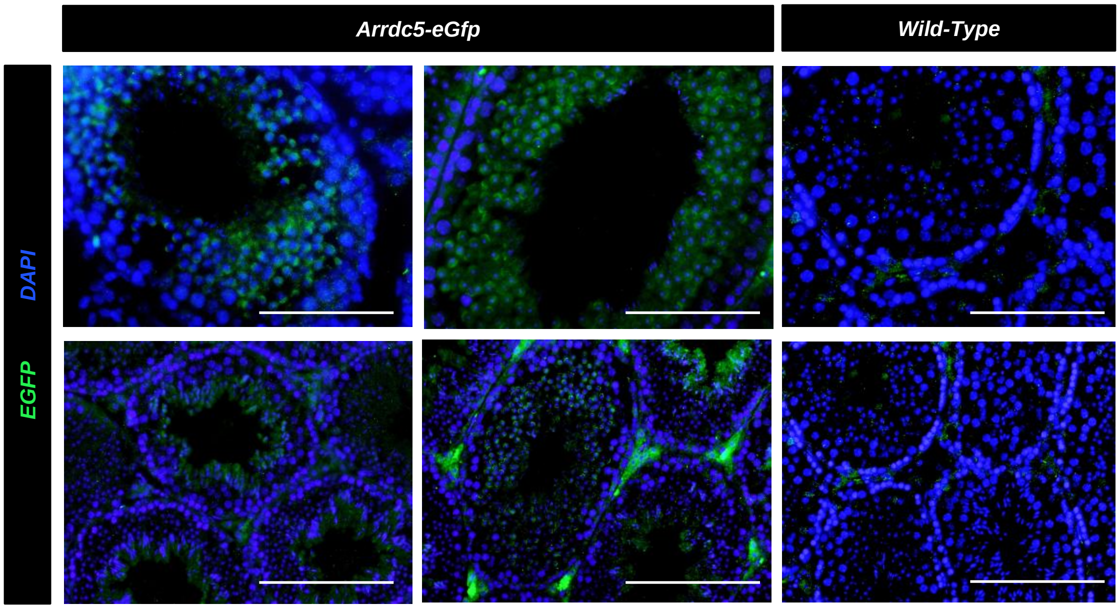

Supplemental Fig. S8: Seminiferous tubules cross-sections from testes of adult *Arrdc5-eGfp* or control mice that were immunostained with an antibody recognizing EGFP. DNA was stained with DAPI. Specific staining for EGFP is observable in round and elongate spermatids of *Arrdc5-eGfp* cross-sections but not detectable in control wild-type cross-sections. Images are representative of 5 independently repeated experiments. Bars are 50 μm in top row and 100 μm in bottom row.

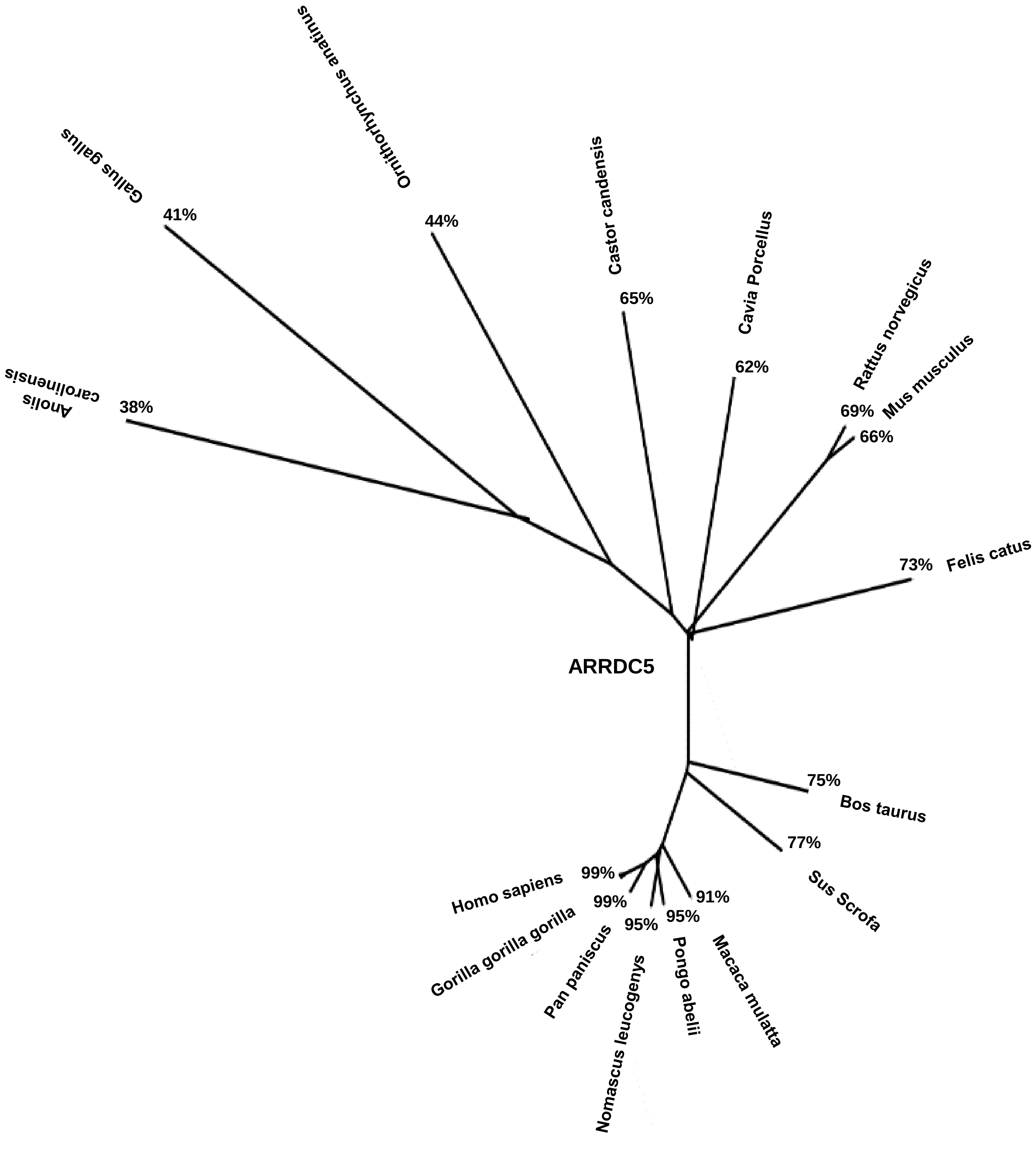

Supplemental Fig. S9: Phylogenetic analysis of ARRDC5 in vertebrate species using Randomized Axelerated Maximum Likelihood. The percent similarity for each species to human ARRDC5 protein sequence is listed.

a

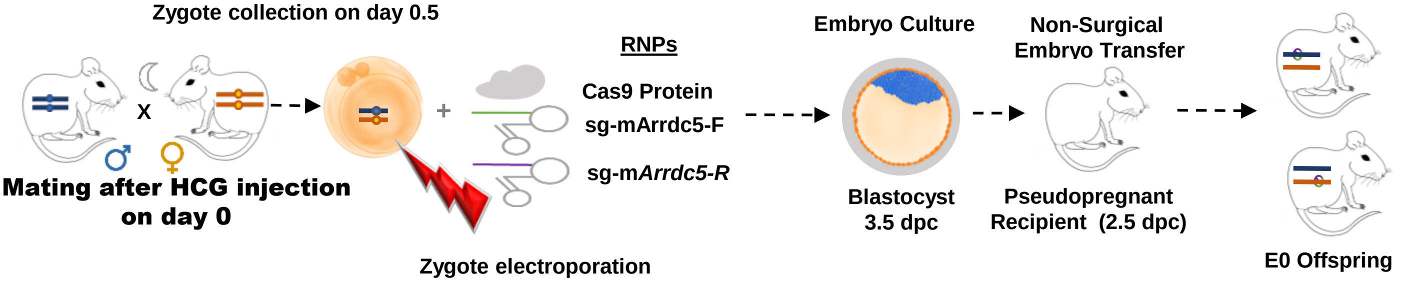

b

Name		5'to3'	PAM	Length
sg-mArrdc5-F	Guide RNA	GGAATCTGGATGATACTCGG	GGG	Δ306
sg-mArrdc5-R	Guide RNA	AAAGGTAAGCATTCGGCCTG	GGG	Δ306
mArrdc5-455F	PCR Genotyping Primer	AACATTGGGTGGAGCGATGT	-	455
mArrdc5-455R	PCR Genotyping Primer	CCCTCTGCCTATCTCTAACTCG	-	455

c

Alignment of Sequence_1: Mus Musculus Wild-Type Arrdc5 Exon 1
Sequence_2: E0 CRISPR Founder Male
Deletion: 308bp (exon 1)

```
SEQ_1 1     AACATTGGGTGGAGCGATGTCACAGAGGACACCACAGCTGCAGAACCCAGGGGAATCTGG 60
                                            | ||  | ||   | |      |||||||||||||
SEQ_2 1     -------------------------------------------C-CC-GCCGCCACCG------GGGGAATCTGG 24

SEQ_1 61    ATGATACTCGGGGGATGGGGAGGATGAGTCTCCCCCGCCCAATCTCCCATGTCTGTGGTG 120
            ||||||||||
SEQ_2 25    ATGATACT---------------------------------------------------- 32

                                          ...

SEQ_1 301   GATGTTATTTGCAATAATAAGGCAGATTACGTACATAAAACAAAAACATTCCCAATAAAA 360

SEQ_2 33    ------------------------------------------------------------ 32

SEQ_1 361   GGTAAGCATTCGGCCTGGGGTGGGAAAGTAAATCCATCTACTGGTCAATCAACAATCTCT 420
                            ||||||||||||||||||||||||||||||||||||||||||||
SEQ_2 33    ----------------GGGGTGGGAAAGTAAATCCATCTACTGGTCAATCAACAATCTCT 76

SEQ_1 421   TAGGAATATCATGCGAGTTAGAG-ATAGGCAGAGGG 431
            |||||||||||||||||||||||| ||||||||||||
SEQ_2 77    TAGGAATATCATGCGAGTTAGAGGATAGGCAGAGGG 112
```

d

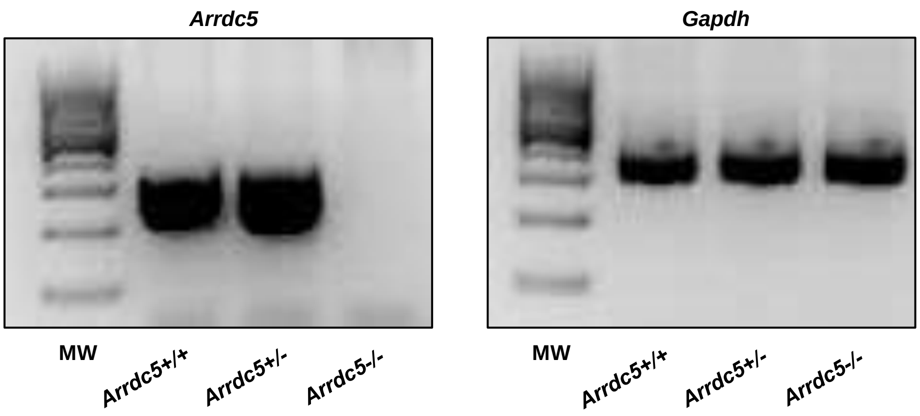

e

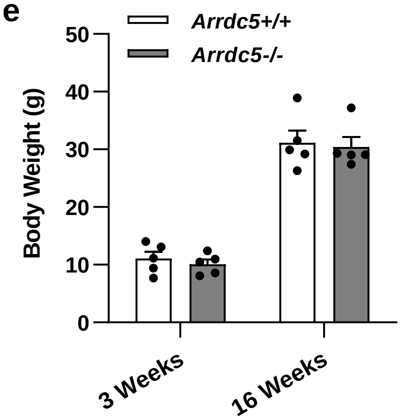

Supplemental Fig. S10: Generation of an *Arrdc5* knockout mouse line. a Schematic of the CRISPR-Cas9 workflow for generation of founder mice with inactivating mutations in the *Arrdc5* gene. **b** Table of single guide RNAs (sgRNAs) sequences targeting the mouse *Arrdc5* coding sequence and genotyping primer sequences. **c** Outcomes of DNA genotyping analysis for a founder male with a 308 bp Δ in exon 1 of the *Arrdc5* coding sequence. **d** Agarose gel visualization of RT-PCR analysis to detect *Arrdc5* mRNA in testes of adult mice that are heterozygous or homozygous for the 308 bp Δ allele, or wild-type (*Arrdc5*+/+). MW is a 100 bp ladder and *Gapdh* was used as a quality control. Image is representative of 3 independently repeated experiments. **e** Quantitative comparison of body weight for adult *Arrdc5-/-* and *Arrdc5*+/+ littermates at 3 and 16 weeks of age. Data bars are mean±SEM for n=5 different males for each genotype at each age. Source data are provided as a Source Data file.

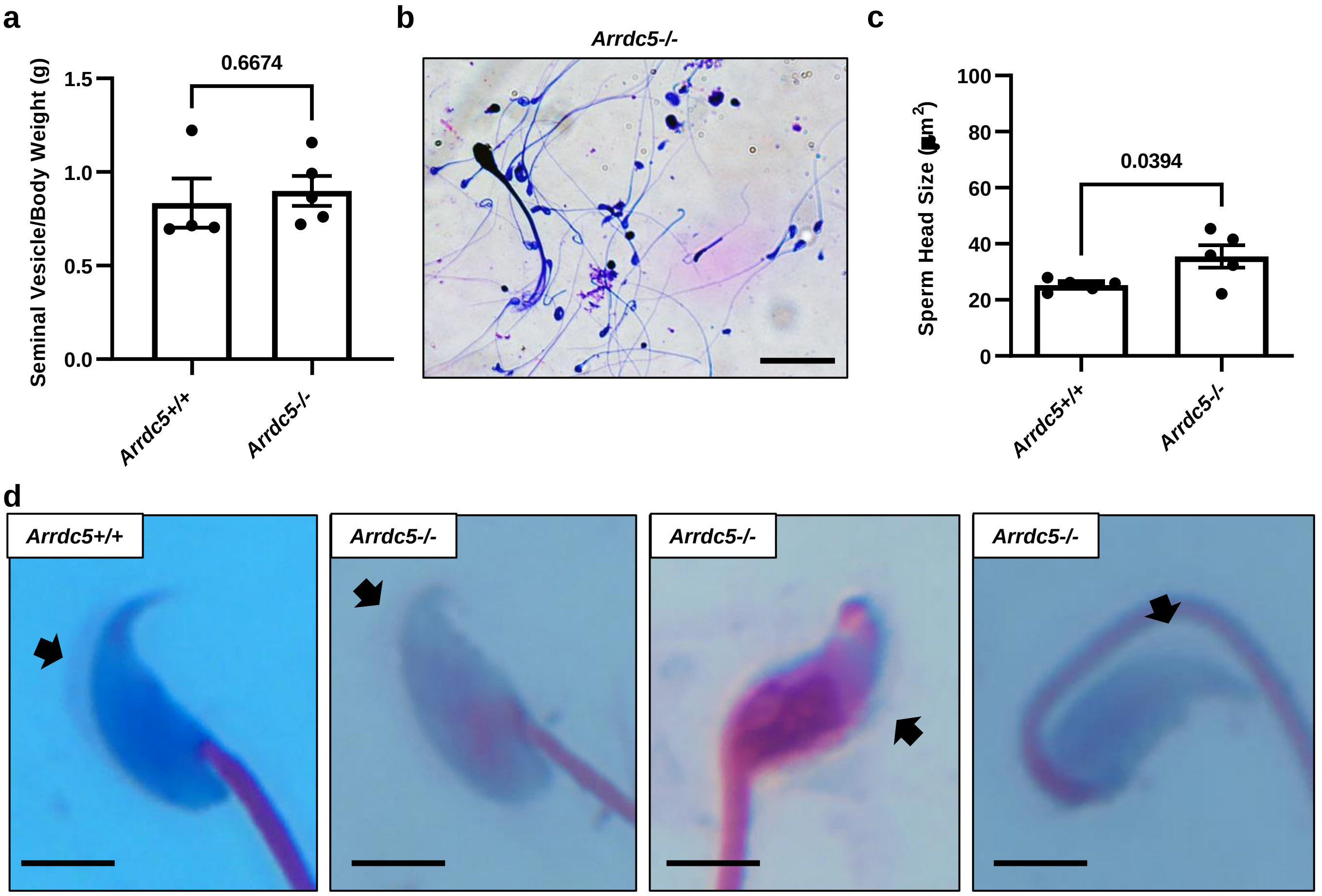

Supplemental Fig. S11: Additional reproductive parameter assessments for *Arrdc5-/-* mice. a Quantitative comparison of the seminal vesicle to body weight ratio of adult *Arrdc5+/+* and *Arrdc5-/-* littermates. Data bars are mean±SEM and dots represent values for individual animals (n=4 for each genotype). **b** Dip Quick stained epididymal sperm from adult *Arrdc5-/-* mice. Scale bar is 10 µm. **c** Quantitative comparison of sperm head size from adult *Arrdc5+/+* and *Arrdc5-/-* littermates. Data bars are mean±SEM and dots represent values for individual animals (n=5 for each genotype). **d** Hematoxylin and eosin-stained sperm heads from adult *Arrdc5+/+* and *Arrdc5-/-* mice. Arrows indicate acrosomes Scale bars are 5 µm. Images in **b** and **d** are representative of 3 independently repeated experiments. Source data are provided as a Source Data file.

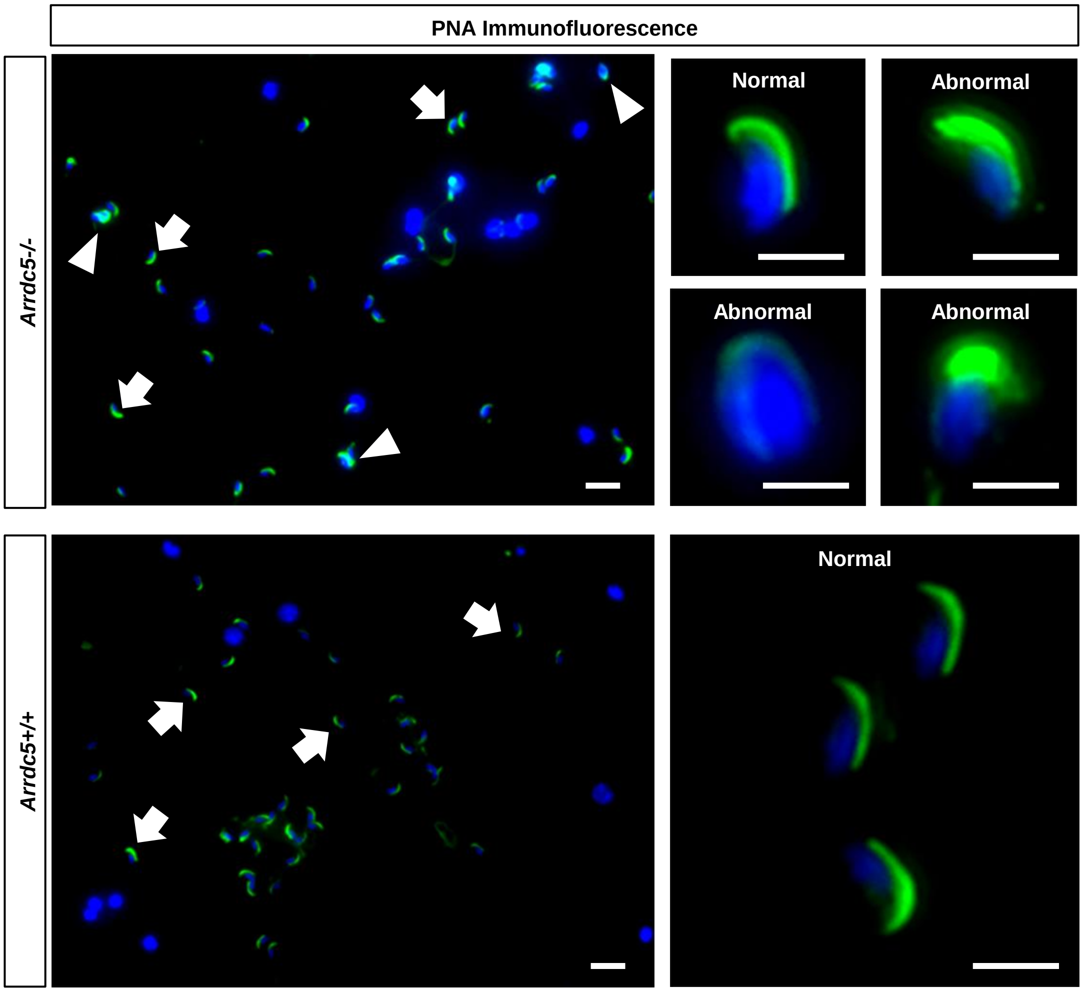

Supplemental Fig. S12: Assessment of acrosome morphology by PNA binding. Epididymal sperm heads from *Arrdc5+/+* and *Arrdc5-/-* littermates were incubated with FITC-conjugated (green) PNA which binds to unreacted acrosomal vesicles and imaged by fluorescent microscopy. DAPI (blue) was used to stain DNA. Bars are 10 μm. Images are representative of 3 independently repeated experiments.

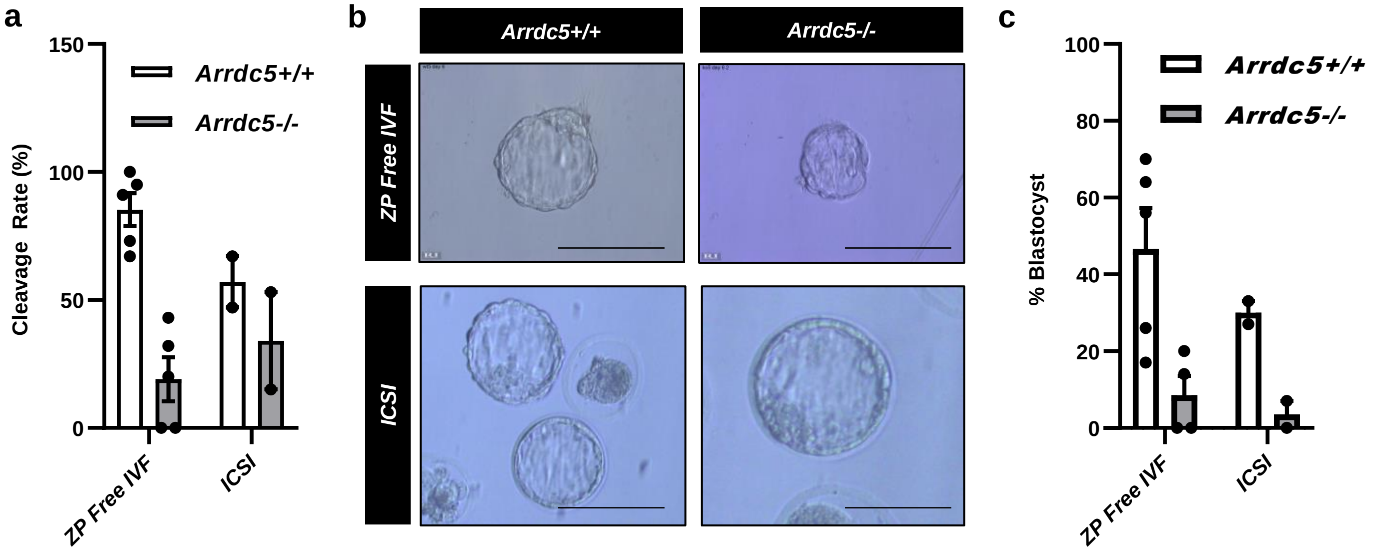

Supplemental Fig. S13: Zona pellucida free *in vitro* fertilization analysis for sperm from *Arrdc5-/-* mice. a Quantitative comparison of embryo cleavage rate following *in vitro* fertilization (IVF) of zona pellucida (ZP) free or intracytoplasmic microinjected (ICSI) wild-type oocytes with epididymal sperm from adult *Arrdc5+/+* or *Arrdc5-/-* littermates. Data bars are mean±SEM and dots represent values for individual animals (IVF: n=5 for each genotype; ICSI: n=2 for each genotype). **b** Blastocyst stage embryos that were generated *in vitro* from ZP free IVF or ICSI with epididymal sperm of adult *Arrdc5+/+* or *Arrdc5-/-* littermates. Images are representative of 5 (IVF) or 2 (ICSI) independently repeated experiments. Bars are 50 μm. **c** Quantitative comparison of *in vitro* blastocyst development rate following IVF of ZP free or ICSI wild-type oocytes with epididymal sperm from adult *Arrdc5+/+* or *Arrdc5-/-* littermates. Data bars are mean±SEM and dots represent values for individual animals (IVF: n=5 for each genotype; ICSI: n=2 for each genotype). Source data are provided as a Source Data file.

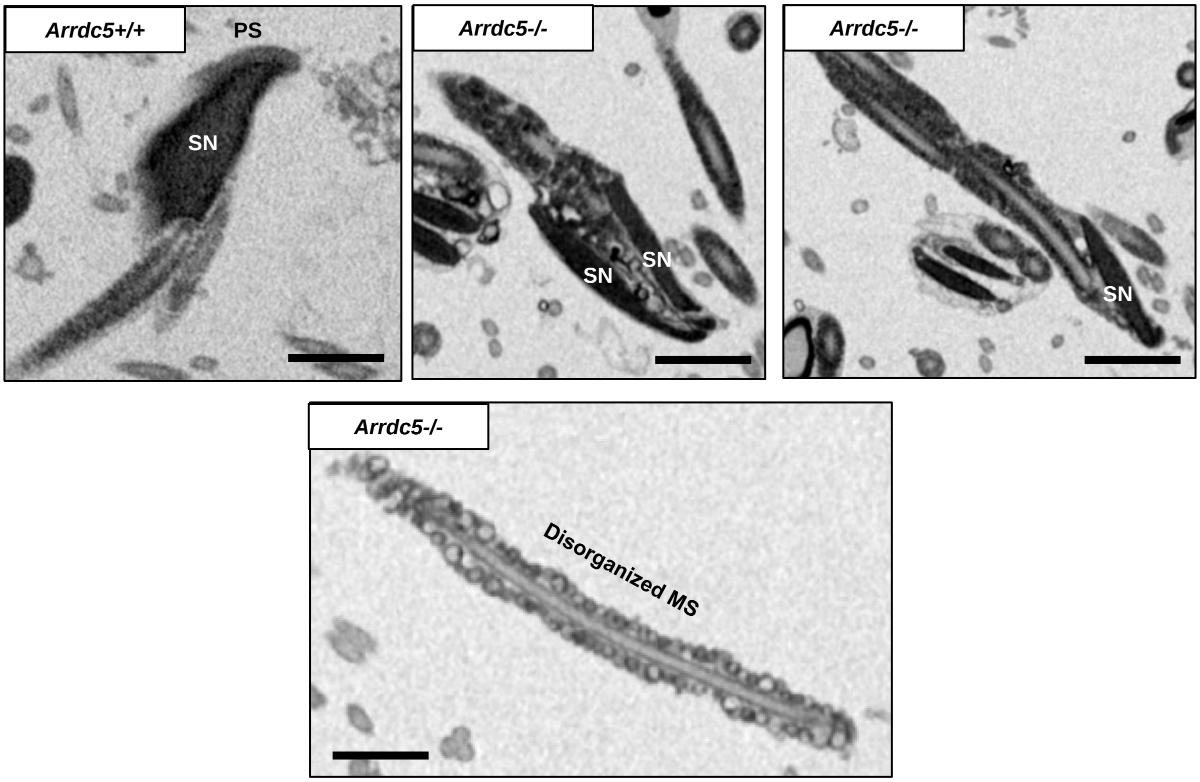

Supplemental Fig. S14: Additional transmission electron microcopy (TEM) images of epididymal sperm from *Arrdc5+/+* and *Arrdc5-/-* littermates. PS = postacrosomal segment, SN = sperm nucleus, and MS = mitochondrial sheath. Bars are 5 μm. Images are representative of 3 independently repeated experiments.

**Supplemental Table S1.** List of key resources and reagents used in this study.		
**Reagents and Resource**	**Source**	**Identifier**
**Antibodies & Probes**		
Alexa Fluor 488 donkey anti mouse IgG (1:200)	Invitrogen, Carlsbad, CA	A21202
Alexa Fluor 488 donkey anti rabbit IgG (1:200)	Invitrogen, Carlsbad, CA	A21206
Alexa Fluor 546 donkey anti mouse IgG (1:200)	Invitrogen, Carlsbad, CA	A10036
Alexa Fluor 546 donkey anti rabbit IgG (1:200)	Invitrogen, Carlsbad, CA	A10040
Rabbit anti Arrdc5 polyclonal (1:200)	Thermo Fisher Scientific, Waltham, MA	PA5-71704
Mouse anti Ddx4 polyclonal (1:150)	Abcam, Cambridge, UK	ab27591
Lectin - PNA Alexa Fluor 488	Invitrogen, Carlsbad, CA	L21409
Normal Mouse IgG (1:200)	Santa Cruz Biotechnology, Dallas, TX	sc-2025
Normal Rabbit IgG (1:200)	Santa Cruz Biotechnology, Dallas, TX	sc2027
Rabbit anti GFP polyclonal (1:250)	Abcam, Cambridge, UK	ab290
**Oligonucleotides**		
#1 Arrdc5 sgRNA (Δ306) PAM GGG F	Integrated DNA Technology, Coralville, Iowa	GGAATCTGGATGATACTCGG
#2 Arrdc5 sgRNA (Δ306) PAM GGG R	Integrated DNA Technology, Coralville, Iowa	AAAGGTAAGCATTCGGCCTG
#3 mus-Arrdc5 gDNA 455bp (genotyping) F	Integrated DNA Technology, Coralville, Iowa	AACATTGGGTGGAGCGATGT
#4 mus-Arrdc5 gDNA 455bp (genotyping) R	Integrated DNA Technology, Coralville, Iowa	CCCTCTGCCTATCTCTAACTCG
#5 mus-Arrdc5 cDNA exon1 435bp (RT-PCR) F	Integrated DNA Technology, Coralville, Iowa	GCCAGAGGAATCTAAGTGAGAACT
#6 mus-Arrdc5 cDNA exon1 435bp (RT-PCR) R	Integrated DNA Technology, Coralville, Iowa	ACAAAGACCAGGGGAGGTGA
#9 bos-Arrdc5 cDNA 249bp (RT-PCR) F	Integrated DNA Technology, Coralville, Iowa	TTAGTGCTGCCCAAGGATGC
#10 bos-Arrdc5 cDNA 249bp (RT-PCR) R	Integrated DNA Technology, Coralville, Iowa	GCCTGCACTTAACCAATTATCCTC
#11 sus-Arrdc5 cDNA 541bp (RT-PCR) F	Integrated DNA Technology, Coralville, Iowa	AAAACCCATCGCAGATTCCTCT

**Supplemental Table S1.** List of key resources and reagents used in this study (continued).		
**Reagents and Resource**	**Source**	**Identifier**
**Oligonucleotides (continued)**		
#12 sus-Arrdc5 cDNA 541bp (RT-PCR) R	Integrated DNA Technology, Coralville, Iowa	TCTGACGATCCATCTGGGCT
#13 mus-Gapdh cDNA 382 bp (RT-PCR) F	Integrated DNA Technology, Coralville, Iowa	AACTTTGGCATTGTGGAAGGGCTC
#12 mus-Gapdh cDNA 382bp (RT-PCR) R	Integrated DNA Technology, Coralville, Iowa	TGGAAGAGTGGGAGTTGCTGTTGA
#14 bos/sus-Gapdh cDNA 388bp (RT-PCR) F	Integrated DNA Technology, Coralville, Iowa	GTGAACGGATTTGGCCGC
#15 bos/sus-Gapdh cDNA 388bp (RT-PCR) R	Integrated DNA Technology, Coralville, Iowa	GGTTCACGCCCATCACAAAC
#18 Arrdc5-egfp sgRNA F	Eurofins Genomics, Louisville, Kentucky	ATGCAGGCACTCAAACCATA
#19 Arrdc5-egfp sgRNA R	Eurofins Genomics, Louisville, Kentucky	GCTGCCATATGAGGACCATA
#20 Arrdc5-egfp LHA F (cloning)	Eurofins Genomics, Louisville, Kentucky	TATCGATAAGCTTGATATCGAGTGCTGACATTGAAGGT
#21 Arrdc5-egfp LHA R (cloning)	Eurofins Genomics, Louisville, Kentucky	AGGTCCAGGGTTCTCCTCCACGTCTCCAGCCTGCTTCAGCAGGCTGAAGTTAGTAGCTCCGCTTCCAACCATGTGATCCCATATG
#22 Arrdc5-egfp EGFP F (cloning)	Eurofins Genomics, Louisville, Kentucky	GGAAGCGGAGCTACTAACTTCAGCCTGCTGAAGCAGGCTGGAGACGTGGAGGAGAACCCTGGACCTATGGTGAGCAAGGGCGAG
#23 Arrdc5-egfp EGFP R (cloning)	Eurofins Genomics, Louisville, Kentucky	CAGGCACTCACTATTACTTGTACAGCTCGTCCATG
#24 Arrdc5-egfp RHA F (cloning)	Eurofins Genomics, Louisville, Kentucky	CAAGTAATAGTGAGTGCCTGCATATTAAATATG
#25 Arrdc5-egfp RHA R (cloning)	Eurofins Genomics, Louisville, Kentucky	TGGATCCCCCGGGCTGCAGGTGCTGCTGTCCTAGTGAC
#26 Arrdc5-egfp LF1 (genotyping/screening)	Eurofins Genomics, Louisville, Kentucky	TTCCTGGGAGCTGAAACGTC
#27 Arrdc5-egfp LF2 (genotyping/screening/HMA)	Eurofins Genomics, Louisville, Kentucky	CACCTCCCCTGGTCTTTGTC
#28 Arrdc5-egfp LR (genotyping/screening)	Eurofins Genomics, Louisville, Kentucky	AAGTCGTGCTGCTTCATGTG
#29 Arrdc5-egfp RR2 (HMA)	Eurofins Genomics, Louisville, Kentucky	GGCTTTGTACATCCCAGGCT
