# Supplementary material for: ARRDC5 expression is conserved in mammalian testes and required for normal sperm morphogenesis
Source: Nat Commun. 2023 Apr 17;14:2111. doi: 10.1038/s41467-023-37735-y (PMC10110545; doi:10.1038/s41467-023-37735-y)
Supplement: Supplementary file 3 — Description of Additional Supplementary Files [file 41467_2023_37735_MOESM3_ESM.pdf]

Description of Additional Supplementary Files

File Name: Supplementary Data 1
Description: Lists of genes from bioinformatic analysis of multispecies testicular single cell transcriptome profiling.
