# Supplementary material for: ARRDC5 expression is conserved in mammalian testes and required for normal sperm morphogenesis
Source: Nat Commun. 2023 Apr 17;14:2111. doi: 10.1038/s41467-023-37735-y (PMC10110545; doi:10.1038/s41467-023-37735-y)
Supplement: Supplementary file 5 — Reporting Summary [file 41467_2023_37735_MOESM5_ESM.pdf]

nature portfolio

Corresponding author(s): Jon Oatley
Last updated by author(s): Mar 4, 2023

Reporting Summary

Nature Portfolio wishes to improve the reproducibility of the work that we publish. This form provides structure for consistency and transparency in reporting. For further information on Nature Portfolio policies, see our Editorial Policies and the Editorial Policy Checklist.

Statistics

For all statistical analyses, confirm that the following items are present in the figure legend, table legend, main text, or Methods section.

n/a	Confirmed	
☐	☒	The exact sample size (*n*) for each experimental group/condition, given as a discrete number and unit of measurement
☐	☒	A statement on whether measurements were taken from distinct samples or whether the same sample was measured repeatedly
☐	☒	The statistical test(s) used AND whether they are one- or two-sided<br>*Only common tests should be described solely by name; describe more complex techniques in the Methods section.*
☒	☐	A description of all covariates tested
☒	☐	A description of any assumptions or corrections, such as tests of normality and adjustment for multiple comparisons
☐	☒	A full description of the statistical parameters including central tendency (e.g. means) or other basic estimates (e.g. regression coefficient) AND variation (e.g. standard deviation) or associated estimates of uncertainty (e.g. confidence intervals)
☐	☒	For null hypothesis testing, the test statistic (e.g. *F*, *t*, *r*) with confidence intervals, effect sizes, degrees of freedom and *P* value noted<br>*Give P values as exact values whenever suitable.*
☒	☐	For Bayesian analysis, information on the choice of priors and Markov chain Monte Carlo settings
☒	☐	For hierarchical and complex designs, identification of the appropriate level for tests and full reporting of outcomes
☒	☐	Estimates of effect sizes (e.g. Cohen's *d*, Pearson's *r*), indicating how they were calculated

Our web collection on statistics for biologists contains articles on many of the points above.

Software and code

Policy information about availability of computer code

Data collection	Sperm parameter data was collected by computer assisted sperm analysis using SCA software (v1.mouse, Microptic)
Data analysis	Microsoft excel version 2202 and GraphPad Prism versions 9.4.0 and 9.4.1 were used to analyze quantitative data. For single-cell RNA-seq (scRNA-seq), raw sequencing reads were demultiplexed and aligned to the mouse genome using the 10X Genomics Cell Ranger pipeline (v2.1.0). scRNA-seq data was analyzed using R software (v3.4.4) with Seurat (v2.3.2) and Monocle (v2.6.4) packages. Gene Ontology was performed with PANTHER (v13.1) software. Sperm DNA integrity was assessed using COMET assay and Comet-Score software (v1.5)

For manuscripts utilizing custom algorithms or software that are central to the research but not yet described in published literature, software must be made available to editors and reviewers. We strongly encourage code deposition in a community repository (e.g. GitHub). See the Nature Portfolio guidelines for submitting code & software for further information.

Data

Policy information about availability of data

All manuscripts must include a data availability statement. This statement should provide the following information, where applicable:

- Accession codes, unique identifiers, or web links for publicly available datasets
- A description of any restrictions on data availability
- For clinical datasets or third party data, please ensure that the statement adheres to our policy

Animal models generated by this study are available through requests of the corresponding author. The single-cell RNA-seq data generated in this study have been

deposited in the Gene Expression Omnibus repository under accession ID GSE206156 (https://www.ncbi.nlm.nih.gov/geo/query/acc.cgi?acc=GSE206156). Source data required for reanalysis are provided with this paper.

Raw base call files from single cell RNA-seq were demultiplexed using the 10X-Genomics Cell Ranger pipeline (v2.1.0) and aligned to publicly available genomes for bovine (ARS-UCD1.2), porcine (Sscrofa11.1), and murine (GRCm39).

Human research participants

Policy information about studies involving human research participants and Sex and Gender in Research.

Reporting on sex and gender	N/A
Population characteristics	N/A
Recruitment	N/A
Ethics oversight	N/A

Note that full information on the approval of the study protocol must also be provided in the manuscript.

Field-specific reporting

Please select the one below that is the best fit for your research. If you are not sure, read the appropriate sections before making your selection.

☒ Life sciences ☐ Behavioural & social sciences ☐ Ecological, evolutionary & environmental sciences

For a reference copy of the document with all sections, see nature.com/documents/nr-reporting-summary-flat.pdf

Life sciences study design

All studies must disclose on these points even when the disclosure is negative.

Sample size	Sample sizes for each experiment were determined using power calculation (beta=0.8) for determining a statistical significance (P<0.05) of 10% or greater difference between mean values.
Data exclusions	No data was excluded from this study.
Replication	Reproducibility was addressed by including at least three biological replicates for each data point collected. All experimental replications were successful.
Randomization	Animals were allocated to experimental groups based on Arrdc5 genotype (i.e. wild-type or Arrdc5-/-). Within a genotype group, individual animals were selected at random for use in experimentation.
Blinding	For mouse studies, investigators were blinded to animal genotype grouping when collecting data on sperm and other phenotype parameters. For other species, only wild-type animals were used, blinding was not necessary.

Reporting for specific materials, systems and methods

We require information from authors about some types of materials, experimental systems and methods used in many studies. Here, indicate whether each material, system or method listed is relevant to your study. If you are not sure if a list item applies to your research, read the appropriate section before selecting a response.

Materials & experimental systems

n/a	Involved in the study	
☐	☒	Antibodies
☒	☐	Eukaryotic cell lines
☒	☐	Palaeontology and archaeology
☐	☒	Animals and other organisms
☒	☐	Clinical data
☒	☐	Dual use research of concern

Methods

n/a	Involved in the study	
☒	☐	ChIP-seq
☒	☐	Flow cytometry
☒	☐	MRI-based neuroimaging

Antibodies

Antibodies used	Rabbit anti Arrdc5 polyclonal, Thermo Fisher Scientific, Waltham, MA, PA5-71704<br>Mouse anti Ddx4 polyclonal, Abcam, Cambridge, UK, ab27591<br>Alexa Fluor 488 donkey anti mouse IgG, Invitrogen, Carlsbad, CA, A21202<br>Alexa Fluor 488 donkey anti rabbit IgG, Invitrogen, Carlsbad, CA, A21206<br>Alexa Fluor 546 donkey anti mouse IgG, Invitrogen, Carlsbad, CA, A10036<br>Alexa Fluor 546 donkey anti rabbit IgG, Invitrogen, Carlsbad, CA, A10040<br>Rabbit anti GFP polyclonal, Abcam, Cambridge, UK, AB290
Validation	Species reactivity and applications for antibodies were validated by the commercial manufacturers and/or previous studies. Relevant information on antibody generation and validation is available at the supplier websites.<br><br>Rabbit anti Arrdc5 polyclonal, Thermo Fisher Scientific, PA5-71704<br>https://www.thermofisher.com/antibody/product/ARRDC5-Antibody-Polyclonal/PA5-71704<br><br>Mouse anti Ddx4 polyclonal, Abcam, ab27591<br>https://www.abcam.com/products/primary-antibodies/ddx4--mvh-antibody-mabcam27591-ab27591.html<br>Zagore LL et al. 2018, Cell Rep 25:1225-1240; Bowles J et al. 2018, Cell Rep 24:1330-1341.<br><br>Alexa Fluor 488 donkey anti mouse IgG, Invitrogen, Carlsbad, CA, A21202<br>https://www.thermofisher.com/antibody/product/Donkey-anti-Mouse-IgG-H-L-Highly-Cross-Adsorbed-Secondary-Antibody-Polyclonal/A-21202<br><br>Alexa Fluor 488 donkey anti rabbit IgG, Invitrogen, Carlsbad, CA, A21206<br>https://www.thermofisher.com/antibody/product/Donkey-anti-Rabbit-IgG-H-L-Highly-Cross-Adsorbed-Secondary-Antibody-Polyclonal/A-21206<br><br>Alexa Fluor 546 donkey anti mouse IgG, Invitrogen, Carlsbad, CA, A10036<br>https://www.thermofisher.com/antibody/product/Donkey-anti-Mouse-IgG-H-L-Highly-Cross-Adsorbed-Secondary-Antibody-Polyclonal/A10036<br><br>Alexa Fluor 546 donkey anti rabbit IgG, Invitrogen, Carlsbad, CA, A10040<br>https://www.thermofisher.com/antibody/product/Donkey-anti-Rabbit-IgG-H-L-Highly-Cross-Adsorbed-Secondary-Antibody-Polyclonal/A10040<br><br>Rabbit anti GFP polyclonal, Abcam, Cambridge, UK, ab290<br>https://www.abcam.com/products/primary-antibodies/gfp-antibody-ab290.html<br>Gong Q et al. 2021 Nat Commun 12:188; Moreno-Ayala R et al. 2021 Cell Rep 34:108606; Vélot L et al. 2021 Mol Cell Proteomics 20:100064; Ferder IC et al. 2019 PLoS Genet 15:e10008084.

Animals and other research organisms

Policy information about studies involving animals; ARRIVE guidelines recommended for reporting animal research, and Sex and Gender in Research

Laboratory animals	Laboratory mice (Mus musculus) used in this study were of mixed 129/C57B6 or CD1/ICR genetic backgrounds. Adult (>P56) males and females were used for experimental analysis.<br><br>Pre-pubertal Holstein breed bull calves at 4.5 months of age and mixed commercial breed pigs at 3 months of age were used as sources multiple tissues.
Wild animals	N/A
Reporting on sex	Although the phenotype of Arrdc5 knockout was male specific sterility, both male and female mice were analyzed for fertility.
Field-collected samples	N/A
Ethics oversight	All animal use in experimentation was approved by the Washington State University Institutional Animal Care and Use Committee.

Note that full information on the approval of the study protocol must also be provided in the manuscript.
